# Brief and Valid? Testing the SDQ for Measuring General Psychopathology in Children

**DOI:** 10.3390/bs15101387

**Published:** 2025-10-13

**Authors:** Victòria Copoví-Gomila, Alfonso Morillas-Romero, Raül López Penadés, María del Àngels Ollers-Adrover, Maria Balle

**Affiliations:** 1 Department of Psychology, University Research Institute on Health Sciences (IUNICS), University of the Balearic Islands, 07122 Palma, Spain; victoriacopovi@hotmail.com (V.C.-G.); maria-dels-angels.ollers@uib.cat (M.d.À.O.-A.); ballemaria@gmail.com (M.B.); 2 Department of Applied Pedagogy and Educational Psychology, Institute of Research and Innovation in Education (IRIE), University of the Balearic Islands, 07122 Palma, Spain; raul.lopez@uib.es

**Keywords:** p factor, Strengths and Difficulties Questionnaire, child psychopathology, bifactor model, CBCL

## Abstract

**Background:** The general psychopathology factor (p factor) is central to understanding the shared variance across mental disorders, offering a dimensional alternative to traditional diagnostic models. The early identification of this factor in childhood is key for improving prevention and intervention strategies. This study evaluated the Strengths and Difficulties Questionnaire (SDQ) as a brief measure to assess p factor in children. **Methods:** A community sample of 284 children, ages 6 to 12, was assessed using parent-reported SDQ and the Child Behavior Checklist (CBCL). Confirmatory Factor Analyses compared two models of psychopathology: a higher-order model and a first-order bifactor model. **Results:** Results showed that the bifactor model provided a better fit for both instruments, with the SDQ showing particularly strong fit indices. Moreover, SDQ-derived p factor scores were strongly correlated with key CBCL scales, particularly attention and externalizing problems, supporting its concurrent validity. **Conclusions:** These findings suggest that the SDQ, due to its brevity and psychometric robustness, is a valid alternative to the CBCL for assessing general psychopathology in children.

## 1. Introduction

The categorical approach to the classification of mental disorders, which classifies psychiatric disorders as distinct and independent entities, has faced substantial criticism in recent years (Eaton et al., 2023; Kapadia et al., 2020; Lilienfeld & Treadway, 2016). In this regard, alongside the ICD-11, the prevailing standard of nosology in clinical psychology and psychiatry remains the Diagnostic and Statistical Manual of Mental Disorders (5th ed.; DSM-5; American Psychiatric Association, 2013). However, growing evidence suggests that these categorical systems fail to align with patients’ clinical presentations (Eaton et al., 2023; Kapadia et al., 2020). Furthermore, dissatisfaction seems to stem from (1) the apparent arbitrariness of diagnostic thresholds, as many disorders appear to share common dimensions; (2) the considerable heterogeneity in clinical manifestations within the same disorder; and, most notably, (3) the high rates of comorbidity and overlap between disorders (Lilienfeld & Treadway, 2016).

Focusing on the latter, these high comorbidity rates suggest that psychopathology may follow a more parsimonious structure than that proposed by nosological systems (Caspi & Moffitt, 2018; Laceulle et al., 2015; Lahey et al., 2012). In this regard, Krueger et al. (1998) proposed a two-factor model of psychopathology that would explain comorbidity between disorders, suggesting the existence of two underlying dimensions instead of categorical clusters: internalizing and externalizing. The first dimension (i.e., internalizing) would be associated with a propensity to develop mood and anxiety disorders, such as generalized anxiety disorder, panic disorder, social phobia, or major depression (Krueger et al., 1998). The second dimension (i.e., externalizing) reflects a liability for substance use disorders and antisocial behaviors, including delinquency, aggression, and attention problems (Laceulle et al., 2015). This approach has been supplemented by some authors with a third dimension—thought disorder—accounting for psychotic symptoms, such as dissociation, unusual beliefs, and hallucinations (Wright et al., 2013); this has been extensively studied, leading to the development of various models that classify traditional DSM-5 symptoms within these dimensions (Caspi et al., 2014; Caspi & Moffitt, 2018; Wright et al., 2013).

However, evidence in the field suggests that even these higher-order dimensions may overlap to a considerable degree (Wright et al., 2013), leading to the proposal of a broader superordinate factor—the General Psychopathology Factor (p factor; Caspi & Moffitt, 2018). Although not exempt of controversy (see Watts et al., 2024 for a critical review), the p factor would reflect a stable and generalized susceptibility to develop various forms of psychopathology throughout the lifespan, while also accounting for comorbidity among disorders, the persistence of disorders over time, and symptom severity (Caspi & Moffitt, 2018). This superordinate dimensional structure of psychopathology has also been widely debated, with seemingly conflicting findings regarding the existence of this general psychopathology factor, either as a replacement for, or in addition to, more specific dimensions (Laceulle et al., 2015).

In this regard, several models have emerged aiming to capture the hierarchical structure of psychopathology and explore its components (Caspi et al., 2024, 2014; Caspi & Moffitt, 2018; De la Cruz et al., 2017; Patalay et al., 2015). In 2018, Caspi and colleagues proposed two models that seemed to better capture the dimensionality of psychopathology: (1) a higher-order or hierarchical model, and (2) a first-order bifactor model. The first model is a higher-order model featuring a second-order factor (the p factor) positioned at a higher level, from which internalizing, externalizing, and psychotic experiences emerge, labeled as first-order latent variables (Caspi et al., 2014; Caspi & Moffitt, 2018). In the second, the general p factor represents a common liability to all forms of psychopathology, alongside a set of independent factors influencing a smaller subset of symptoms and disorders (Caspi & Moffitt, 2018). Even though, in both models, the p factor captures the common variance shared across all symptoms and the latent specific factors reflect the additional shared variance among specific symptoms (e.g., Castellanos-Ryan et al., 2016), no absolute agreement has been reached regarding which model better represents the structure of psychopathology. In fact, a recent study by Caspi et al. (2024) found a strong correlation between both models, indicating that they classify individuals in a similar way along a latent dimension of general psychopathology, highlighting the need for further research in this area. In any case, regardless of which model provides the best fit for evaluating p factor, it is clear that, in order to achieve the expected clinical utility, it must be clearly measurable (Caspi & Moffitt, 2018).

Another way to enhance its clinical utility, in terms of prevention and the detection of potential risk factors for psychopathology, may involve investigating these psychopathological structures and models in earlier developmental stages. Furthermore, expanding research on the p factor to include community samples of children may be crucial for early detection of psychopathology, which is directly associated with better prognosis and a reduced risk of developing additional psychological issues (Alonso et al., 2012).

However, assessing the p factor in children is far from straightforward. In this regard, the gold-standard measure is the Child Behavior Checklist (CBCL), which has demonstrated excellent psychometric properties and includes scales for a wide range of behavioral and emotional problems (Achenbach & Rescorla, 2001). Despite its robust reliability, the comprehensive nature of the instrument necessitates a significant number of items (113), which makes it a time-consuming tool. Consequently, the CBCL becomes a highly demanding measure, highlighting the need for more efficient alternatives that can still capture the broader structure of psychopathology. Such alternatives would not only reduce the burden on respondents but also, ultimately, facilitate the development of new studies that could help clarify which model (i.e., the hierarchical model or the first-order bifactor model proposed by Caspi & Moffitt, 2018) better represents the structure of psychopathology.

In this regard, a promising and shorter alternative to the CBCL for evaluating the p factor in children appears to be the Strengths and Difficulties Questionnaire (SDQ; Goodman, 1997). The SDQ has demonstrated potential in assessing the p factor and effectively captures emotional and behavioral problems in children and adolescents (De la Cruz et al., 2017; Patalay et al., 2015). It has been shown to have high correlation with the CBCL, as both instruments yield scores for Internalizing, Externalizing, and Total Problems (Goodman & Scott, 1999). Given these similarities, it seems logical to propose the SDQ as a potential tool for assessing the psychopathological models presented by Caspi and Moffitt (2018). However, studies employing this questionnaire to assess these models remain limited and results are inconsistent (Afzali et al., 2017; Carragher et al., 2015; De la Cruz et al., 2017), highlighting the need of further research.

Based on the aforementioned considerations, the main aim of the present study was to examine the validity of SDQ as a measure of the p factor in a community sample of children. Confirmatory Factor Analyses (CFAs) were conducted to assess the fit of two competing models of psychopathology: a higher-order model (Model 1) and a first-order bifactor model (Model 2). As a secondary objective, we aimed to evaluate the concurrent validity of the p factor scores derived from the best-fitting SDQ model by conducting correlational analyses with the CBCL scales.

## 2. Methods

### 2.1. Participants

Parents from a total of 284 children entered the study (87.7% mothers). Children were boys and girls aged 6 through 12 (*M* = 9.13; *SD* = 1.78; 41.9% girls). No psychological or sociodemographic information other than age was considered when determining participants’ eligibility.

### 2.2. Procedure

A total of 57 primary schools were contacted. From the pool of approximately 300 primary schools in the Balearic Islands (Spain), and in accordance with a cluster sampling design, schools were randomly selected and contacted by telephone to inform them about the purpose of the study. The participating schools included both public and private institutions, located in urban and suburban areas. Details about the study and the information to be collected were first sent via mail, and then disseminated through the official academic management platform used for communication between families and schools. All families with children enrolled in grades 1 to 6 of primary education received this communication. Before self-reported measures were collected through online versions of the questionnaires via Google Forms, an Informed Consent was filled. Participants did not receive any financial compensation for their involvement. This study is part of a larger research project approved by a university-affiliated Research Ethics Committee.

### 2.3. Measures

#### 2.3.1. Strengths and Difficulties Questionnaire (SDQ)

The Strengths and Difficulties Questionnaire (SDQ; Goodman, 1997) is a parent-reported, 25-item behavioral screening tool. It is part of the Development and Well-Being Assessment (DAWBA; Goodman et al., 2000) family of mental health measures. It includes five subscales: emotional problems, peer problems, behavioral problems, hyperactivity, and prosocial behavior. Each subscale comprises a total of five questions scored on a three-point Likert-type scale (0 = *Not true*; 1 = *Somewhat true*; 2 = *Certainly true*). From the total of 25 items, ten are reverse-scored if they contribute to the peer, behavioral, hyperactivity or prosocial subscales. This instrument has been validated in Spanish samples (García et al., 2000). Descriptive and reliability statistics are shown in Table 1.

#### 2.3.2. Child Behavior Checklist (CBCL)

The Child Behavior Checklist/6-18 (CBCL) is a 113-item parent-informed measure from the Achenbach System of Empirical Based Assessment (ASEBA; Achenbach & Rescorla, 2001). It is one of the most used parent-report questionnaires in child and adolescent psychiatric research (Achenbach, 1991; Verhulst & Achenbach, 1995). The symptom dimensions covered are Anxious/Depressed, Withdrawn/Depressed, Somatic Complaints, Social Problems, Thought Problems, Attention Problems, Rule-Breaking Behavior, Aggressive Behavior, and Other Problems. All items are scored on a three-point Likert-type scale (0 = *not true*; 1 = *somewhat true*; 2 = *very or often true*). This instrument has been validated in Spanish samples (Sardinero García et al., 1997). Descriptive and reliability statistics are shown in Table 1.

### 2.4. Statistical Analyses

**Figure 1:**
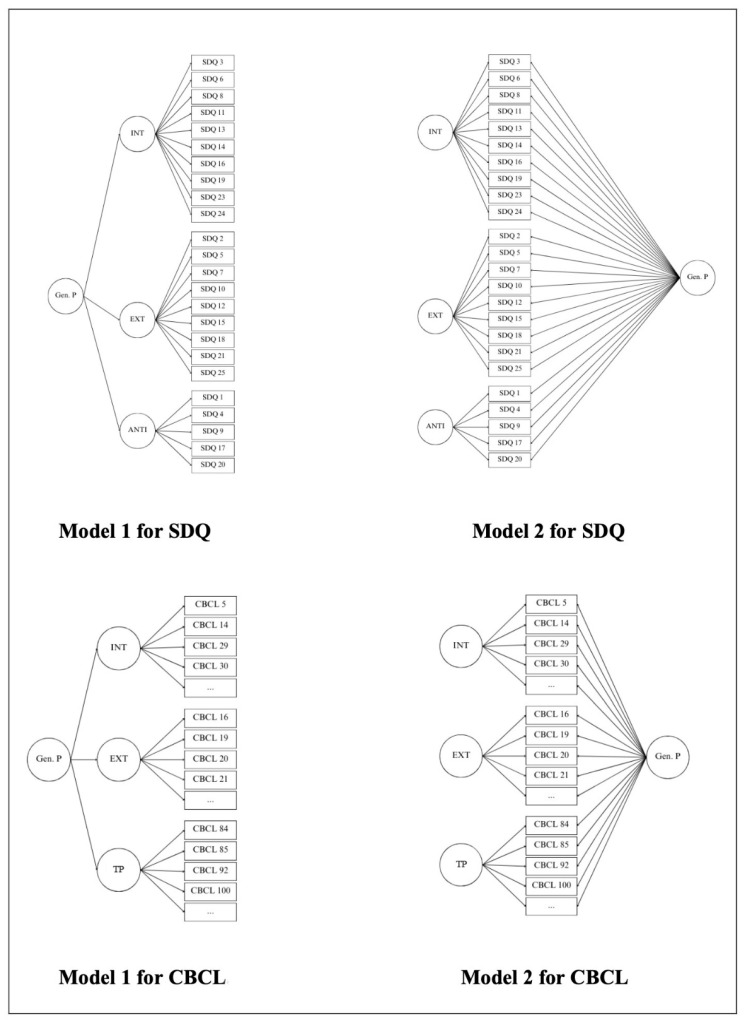
Assessed models for both SDQ and CBCL. *Note. Gen. P* = General Psychopathology Factor; *INT* = Internalizing; *EXT* = Externalizing; *ANTI* = Antisocial Behavior; *TP* = Thought Problems; *SDQ* = Strengths and Difficulties Questionnaire; *CBCL* = Child Behavior Questionnaire.

We used CFA to evaluate and compare the fit of two alternative factor structures (Model 1: higher-order and Model 2: first-order bifactor; see Figure 1) for the parent versions of SDQ and CBCL in our sample. In the case of SDQ, the models included three latent factors: Internalizing with items from the Emotional Problems and Peer Problems scales; Externalizing with items from the Behavioral Problems and Hyperactivity scales (De la Cruz et al., 2017); and Antisocial with reversed items from the Prosocial Behavior scale. For the CBCL three latent factors were also constructed: Internalizing with items from Anxious/Depressed, Withdrawn/Depressed, and Somatic Complaints scales; Externalizing with items from Rule-Breaking Behavior and Aggressive Behavior scales (Achenbach & Rescorla, 2007); and Thought Problems with items from Thought Problems Scale.

We performed the analyses in MPlus version 8.10 by using a multivariate probit analysis for ordinal data and using the Weighted, Least Squares, Mean, and Variance, adjusted (WLSMV) for the estimation of the model fit because of the categorical nature of the variables. The WLSMV is a robust estimator, primarily recommended for CFA involving ordered-categorical data, including dichotomous variables. It does not require the assumption of normally distributed variables and is suggested to be the optimal choice for modeling categorical data in relatively small samples (Beauducel & Herzberg, 2006).

As absolute fit indexes we obtained the chi-square statistic, the relative chi-square (χ^2^/*df*)—adjusting chi-square for sample size—the Standardized Root Mean Square of Residuals (SRMR), and the Root Mean Square Error of Approximation (RMSEA). The Comparative Fit Index (CFI) and the Tucker–Lewis Fit Index (TLI) were obtained as relative measures of fit. Interpretation of fit indexes was based on conservative cutoff values. To consider a model as showing a “good fit”, we required a SRMR < 0.08, RMSEA < 0.06, CFI > 0.95 and TLI > 0.95 (Hu & Bentler, 1999). Under a non-conservative approach, CFI and TLI > 0.90 have been considered as indicators of “acceptable fit” (Brown, 2015). Regarding relative chi-square, it has been proposed that a value smaller than 2 reflects good fit (Ullman, 2001).

In order to assess the concurrent validity of the p factor, we computed correlations between latent p factor scores and CBCL scale scores. Based on participant’s observed responses and the estimated model parameters, MPlus estimates latent factor scores as the maximum of the posterior distribution of the factor, which is also called the Maximum A Posteriori (MAP) method (Muthén & Muthén, 2017). Shapiro-Wilk tests were performed and showed that the distribution of the variables departed significantly from normality (*W*s < 0.98, *p*s < 0.002). Based on this outcome, non-parametric Spearman correlations were selected as they account for deviations from normality assumptions.

## 3. Results

### 3.1. Descriptive Statistics

Table 1 shows descriptive statistics and internal consistency for SDQ and CBCL scales. SDQ scales ranged from moderate to good internal consistency. Most CBCL syndrome scales showed adequate internal consistency. Internal consistency for CBCL broadband scales (i.e., Internalizing and Externalizing) was good.

### 3.2. Confirmatory Factor Analyses (CFA)

It should be noted that when running CFAs some items had to be excluded from the analyses since they had a variance near to 0. This happens in those items where most of the answers are 0 = *Not True*. Thus, for subsequent confirmatory analyses (see section below), item 22 from the SDQ (“*Steals from home, school or elsewhere*”) was excluded due to its very low variability, *M* = 0.02, *SD* = 0.18, which caused negative residual variances (Brown, 2015). This, in turn, precluded the computation of latent factor scores.

Regarding the CBCL, item 2 = “*Drinks alcohol without parents’ approval*”, item 67 = “*Runs away from home*”, item 72 = “*Sets fires*”, item 99 = “*Smokes, chews or sniffs tobacco*”, and item 105 = “*Uses drugs for non-medical purposes*” were also excluded from analyses due to their very low variability. Items 67 and 72 belong to the Rule-Breaking Behavior subscale and the rest, to the Other Problems subscale. These items refer to extreme behaviors that were rated as 0 = Not True, in all the cases.

Once all items mentioned above were excluded, CFAs showed that the first-order bifactor model was the best fitting model for both SDQ and CBCL. However, all fit indices were better for the SDQ in comparison to the same model for the CBCL (see Table 2). Relative Chi-Square, SRMR and RMSEA of the first-order bifactor model for the SDQ resulted in a good fit and CFI and TLI in an acceptable fit. Overall, and taking all the goodness-of-fit indices into account, Model 2 (first-order bifactor model) for SDQ was the one that showed the best fit to the data among all the models tested for both questionnaires.

### 3.3. Concurrent Validity

P factor latent scores from SDQ through model 2 were correlated with CBCL’s scales with an established bond with psychopathology in childhood to examine the associations of general psychopathology measured through SDQ and the specific scales of CBCL.

Results showed that all scales from the CBCL had a positive and significant correlation with the general p factor scores from the SDQ (see Table 3). The scales that resulted in higher correlations were *Attention Problems*, *r* = 0.65, *p* < 0.001; *Aggressive behavior*, *r* = 0.66, *p* < 0.001; and the general *Externalizing*, *r* = 0.69, *p* < 0.001 scale.

**Figure 2:**
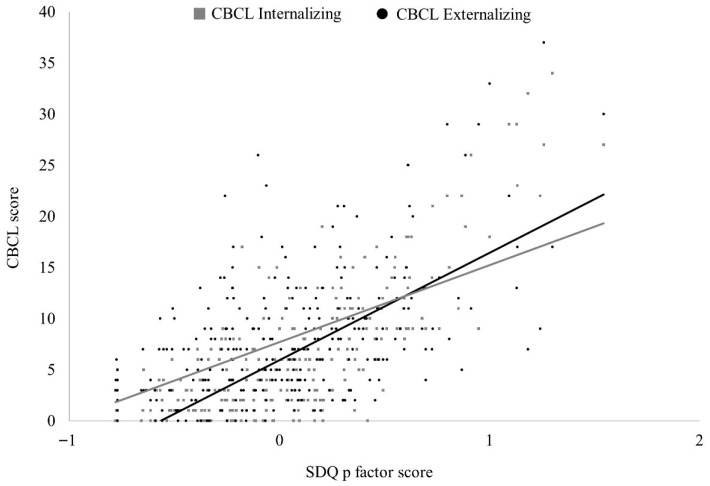
Association between SDQ p factor latent scores and CBCL broad-band scales. *Note*. SDQ = Strengths and Difficulties Questionnaire; CBCL = Child Behavior Checklist.

These results suggest the efficacy of the p factor from SDQ to capture different domains of psychopathology. The correlational pattern of p factor with both, CBCL Internalizing and Externalizing scales (among others) suggests that the computed scores from the latent p factor would represent commonalities among these two dimensions of psychopathology. For illustrative purposes, the association between SDQ p factor latent scores and CBCL broadband scales are depicted in Figure 2.

## 4. Discussion

The main aim of the present study was to examine, through Confirmatory Factor Analyses (CFA), the fit of the higher-order model (Model 1) and the first-order bifactor model (Model 2) of p factor using the SDQ in a community sample of 284 children. Additionally, we sought to evaluate the concurrent validity of the p factor derived from the best-fitting SDQ model by conducting correlational analyses with the CBCL scales.

Based on our results, the first-order bifactor structure demonstrated the best fit for both the SDQ and CBCL, surpassing the higher-order model. Moreover, goodness-of-fit indices indicated that the first-order bifactor model provided a better fit for the SDQ than for the CBCL, that exhibited a poor fit. These findings suggest that the SDQ may serve as a promising and efficient alternative to the CBCL for capturing the p factor. However, few studies have previously examined how different psychopathological models fit for SDQ in children or adolescents, and the results from these studies remain inconsistent (Afzali et al., 2017; Carragher et al., 2015; De la Cruz et al., 2017).

In line with our results, Carragher et al. (2015) and Afzali et al. (2017) identified the modified bifactor model, which included three correlated specific factors (internalizing, externalizing, and thought disorder) and one general psychopathology factor, as the best-fitting model. On the other hand, contrary to our outcomes, De la Cruz et al. (2017) highlighted a first-order five-factor model to be the best solution. Although it may seem somehow contradictory, all results are consistent with those found by Caspi et al. (2024), recently highlighting a strong correlation between both models, indicating that they classify individuals in a similar way along a latent dimension of general psychopathology. However, Caspi et al. (2024) did not use the SDQ or the CBCL to assess p components. Nevertheless, given the inconsistencies in findings and conclusions in this field, assessing the psychopathological structure in children remains a work in progress.

Regarding our secondary aim, all CBCL scales showed significant correlations with general p factor scores assessed through the SDQ, supporting the SDQ’s validity in capturing various domains of psychopathology. These findings align with previous studies demonstrating the adequate concurrent validity of the SDQ in comparison to the CBCL and other clinical psychopathological measures (e.g., Achenbach et al., 2008; Goodman & Scott, 1999; Koskelainen et al., 2000; Laugen et al., 2024; Mansolf et al., 2022). Consistent with our results, Goodman and Scott (1999) found moderate to high correlations between SDQ subscales and their analogous CBCL scales. Furthermore, they observed that the SDQ was significantly more effective in detecting inattention and hyperactivity and at least as effective as the CBCL in identifying both internalizing and externalizing problems. However, the sample age in their study (4–7 years old) was notably younger than that of our study. A similar age difference is noted in the recent study by Laugen et al. (2024), which, using a sample of children aged 2–4 years, found moderate to significant positive correlations between SDQ total scores and CBCL subscales and total score. In contrast, a study conducted in Finland with general population reported moderate to high correlations between parent-reported SDQ and CBCL in children aged 7 to 15 years (Koskelainen et al., 2000), which is more closely aligned with the age range of our sample. Mansolf et al. (2022) examined internalizing, externalizing, and total problems using both the CBCL and SDQ, finding that the measures were highly correlated across three age groups ranging from 2 to 17 years. Finally, and also consistent with our findings, Achenbach et al. (2008) reported comparable results between the SDQ and the CBCL in various populations included in the Achenbach System of Empirically Based Assessment (ASEBA).

Building on our results, this evidence further underscores the SDQ as a valid tool for assessing the psychopathological structure in children and, as previously mentioned, a potentially more time-efficient alternative to the CBCL. It is important to note, as highlighted by Mansolf et al. (2022), that the reduction in item count compared to the CBCL may lead to lower internal consistency. This was partially evidenced in our study where the internal consistency of the SDQ subscales ranged from 0.60 to 0.81, indicating questionable reliability for some subscales while moderate to high reliability for others.

Overall and considering the evidence presented thus far, the SDQ emerges as a highly suitable alternative for assessing the p factor in children, given its strong alignment with the first-order bifactor model and its efficiency.

### Limitations and Future Research

Several limitations of the present study must be considered. First, the sample size in our study, particularly for the CBCL, is relatively small. Wolf et al. (2013) note that general sample size guidelines are challenging to establish, but some benchmarks exist: (a) a minimum of 100–200 participants (Boomsma, 1982, 1985), (b) 5–10 observations per estimated parameter (*N/q* > 5; Bentler & Chou, 1987), and (c) 10 cases per variable (Nunnally, 1967). Our sample of *n* = 284 meets criterion (a) for both SDQ and CBCL. However, criterion (b) is not met, with only 2.98 observations per parameter for the SDQ first-order bifactor model and 3.78 for the higher-order model. Criterion (c) is met for SDQ (11.8 cases per variable), but not for CBCL. While not all criteria are met, the sample size requirements are moderately satisfied, and further research with larger samples is warranted. Second, and related to the previous limitation, we did not test for Measurement Invariance (MI), which could confirm whether the factorial structure holds across gender and age or reflects the trends identified by Patalay et al. (2015), who reported the emergence of a general psychopathology factor in early adolescence. However, due to our sample size, conducting MI analysis would compromise the CFAs due to insufficient participants in each group, potentially undermining statistical significance. Thus, future studies with larger child samples are needed.

The third limitation refers to the number of items in each questionnaire. CFA model fit tends to worsen with a higher number of items (Kenny & McCoach, 2003). Given the considerable difference in item count between SDQ and CBCL, this issue should be considered when interpreting our results about the poor fit of the structural models based on the CBCL. Finally, a further limitation is the lack of detailed sociodemographic data, which limits the possibility of examining their potential influence on the findings.

Future research should focus on analyzing the structure of psychopathology in larger samples, allowing for the examination of possible invariances of age and/or gender on model fit. Longitudinal studies tracking children’s development into adolescence could further clarify the model’s applicability across different age groups. Expanding the literature on the p factor and child psychopathology is essential, as this remains a relatively new area requiring more in-depth exploration. This is especially important given the recent critical review by Watts et al. (2024), which challenged the validity of the p factor as a latent construct, raising concerns about its replicability and its heavy reliance on statistical models. Nonetheless, the authors emphasize the need for exploratory studies and methods, such as those proposed in this work, as promising tools for uncovering new insights into the structure of psychopathology and the p factor.

## 5. Conclusions

Our study found that the bifactor model not only provided superior fit but also yielded valuable insights into child psychopathology. The general p factor, which encapsulates shared variance across items, strongly correlated with overall mental health indicators, supporting its validity as a broad measure of psychopathology. Our results also showed that specific factors, while secondary to the p factor, captured unique aspects of psychopathology crucial for targeted interventions, echoing Stochl et al. (2015). This study contributes to the growing body of evidence supporting the bifactor model in child psychopathology and introduces the SDQ as an efficient tool for this purpose. Furthermore, our research underscores the status of SDQ as a practical and efficient instrument for identifying general psychopathology in children, with significant implications for early detection and prevention strategies that can support more effective interventions and foster better long-term mental health outcomes.

## Figures and Tables

**Table 1:** Descriptive statistics and internal consistency for SDQ and CBCL scales.

	*N* Items	*M*	(*SD*)	*Min.*	*Max.*	Cronbach’s Alpha
**SDQ scales**						
Emotional Problems	5	1.63	(1.66)	0	9	0.60
Peer Problems	5	1.17	(1.53)	0	7	0.62
Behavioral Problems	5	1.07	(1.40)	0	7	0.64
Hyperactivity	5	3.45	(2.57)	0	7	0.81
Prosocial Behavior	5	8.16	(1.97)	1	10	0.78
**CBCL scales**						
Anxious/Depressed	13	4.15	(3.34)	0	17	0.72
Withdrawn/Depressed	8	1.95	(2.45)	0	14	0.77
Somatic Complaints	11	1.85	(1.98)	0	14	0.59
Social Problems	11	2.49	(2.51)	0	12	0.69
Thought Problems	15	2.09	(2.34)	0	15	0.60
Attention Problems	10	4.07	(3.46)	0	16	0.81
Rule-Breaking Behavior	12	1.32	(1.70)	0	9	0.61
Aggressive Behavior	18	4.98	(5.01)	0	27	0.89
Other problems	15	3.33	(2.61)	0	14	0.58
Internalizing	32	7.95	(6.29)	0	37	0.85
Externalizing	30	6.29	(6.30)	0	34	0.89

*Note*. CBCL = Child Behavior Checklist; SDQ = Strengths and Difficulties Questionnaire.

**Table 2:** Model fit in CFA for SDQ and CBCL questionnaires.

	Model	*χ* ^2^	*χ*^2^/df	SRMR	RMSEA	CFI	TLI
SDQ	Model 1	633.70	2.54	0.11	0.07	0.84	0.83
	Model 2	395.89	1.72	0.08	0.05	0.93	0.92
CBCL	Model 1	7503.52	2.6	0.36	0.08	0.45	0.43
	Model 2	6496.17	2.34	0.34	0.07	0.56	0.54

*Note.* Model 1 = higher-order model; Model 2 = first-order bifactor model; *χ*^2^ = Chi-square; *χ*^2^/df = Relative chi-square; SRMR = Standardized Root Mean Square of Residuals; RMSEA = Root Mean Square Error of Approximation; CFI = Confirmatory Fit Index; TLI = Tucker–Lewis Fit Index; SDQ = Strengths and Difficulties Questionnaire; CBCL = Child Behavior Checklist.

**Table 3:** Spearman correlations between p factor and CBCL subscales.

P Factor	CBCL Scale	Spearman Correlation
P	*Anxious/Depressed*	0.47 **
P	*Withdrawn*	0.44 **
P	*Somatic Complaints*	0.23 **
P	*Social Problems*	0.57 **
P	*Thought Problems*	0.44 **
P	*Attention Problems*	0.65 **
P	*Rule-Breaking Behavior*	0.58 **
P	*Aggressive Behavior*	0.66 **
P	*Other Problems*	0.46 **
P	*Internalizing*	0.50 **
P	*Externalizing*	0.69 **

*Note*. * *p* < 0.01; ** *p* < 0.001.

## Data Availability

The data supporting the findings of this study are available from the corresponding author upon reasonable request.
